# Genome-wide association study of antibody response to Newcastle disease virus in chicken

**DOI:** 10.1186/1471-2156-14-42

**Published:** 2013-05-10

**Authors:** Chenglong Luo, Hao Qu, Jie Ma, Jie Wang, Chunyu Li, Chunfen Yang, Xiaoxiang Hu, Ning Li, Dingming Shu

**Affiliations:** 1 Institute of Animal Science, Guangdong Academy of Agricultural Sciences, 1 Dafeng 1st Street, Wushan, Tianhe District, Guangzhou, Guangdong, 510640, China; 2 State Key Laboratory for Agro-Biotechnology, China Agricultural University, Beijing, 100193, China; 3 State Key Laboratory of Livestock and Poultry Breeding, Guangzhou, 510640, China

**Keywords:** Chicken, Newcastle disease, Antibody response, Genome-wide association study

## Abstract

**Background:**

Since the first outbreak in Indonesia in 1926, Newcastle disease has become one of the most common and contagious bird diseases throughout the world. To date, enhancing host antibody response by vaccination remains the most efficient strategy to control outbreaks of Newcastle disease. Antibody response plays an important role in host resistance to Newcastle disease, and selection for antibody response can effectively improve disease resistance in chickens. However, the molecular basis of the variation in antibody response to Newcastle disease virus (NDV) is not clear. The aim of this study was to detect genes modulating antibody response to NDV by a genome-wide association study (GWAS) in chickens.

**Results:**

To identify genes or chromosomal regions associated with antibody response to NDV after immunization, a GWAS was performed using 39,833 SNP markers in a chicken F_2_ resource population derived from a cross between two broiler lines that differed in their resistance. Two SNP effects reached 5% Bonferroni genome-wide significance (*P*<1.26×10^-6^). These two SNPs, *rs15354805* and *rs15355555*, were both on chicken (*Gallus gallus*) chromosome 1 and spanned approximately 600 Kb, from 100.4 Mb to 101.0 Mb. *Rs15354805* is in intron 7 of the chicken *Roundabout*, *axon guidance receptor*, *homolog 2* (*ROBO2*) gene, and *rs15355555* is located about 243 Kb upstream of *ROBO2*. *Rs15354805* explained 5% of the phenotypic variation in antibody response to NDV, post immunization, in chickens. *Rs15355555* had a similar effect as *rs15354805* because of its linkage disequilibrium with *rs15354805* (r^2^=0.98).

**Conclusion:**

The region at about 100 Mb from the proximal end of chicken chromosome 1, including the *ROBO1* and *ROBO2* genes, has a strong effect on the antibody response to the NDV in chickens. This study paves the way for further research on the host immune response to NDV.

## Background

Newcastle disease is a highly contagious viral disease of birds affecting over 250 domestic and wild avian species. Since Newcastle disease was first discovered in Indonesia in 1926, it has spread throughout the world [1]. Newcastle disease is one of the most serious problems affecting poultry industries in many countries because its contagiousness and devastating effects [2-5]. Newcastle disease virus (NDV) has been classified into velogenic, mesogenic and lentogenic strains on the basis of their pathogenesis and virulence. Chickens with Newcastle disease have severe neurological and respiratory signs and show decreased egg quality and production [1,6,7]. There is no effective treatment for Newcastle disease; however, the use of prophylactic vaccines and maintenance of strict biosecurity measures can reduce the likelihood of outbreaks. Thus, the ability of chickens to mount an antibody response to NDV plays a key role in controlling Newcastle disease outbreaks, why understanding the molecular basis of immune response to NDV is important for the control of avian Newcastle disease. The antibody response to the same virus differs between chicken breeds [8], and selection for an antibody response may improve disease resistance in chickens [9]. However, there have been few studies on controlling Newcastle disease outbreaks from the perspective of host resistance. Ten quantitative trait loci (QTLs) have been reported to be linked to the antibody response to NDV on chicken (*Gallus gallus*) chromosomes (GGA) 2, 3, 4, 5, 9, 13, 16, 18, 22 and Z [10,11], but only a few causative genes have been identified because of low map resolution. The chicken 60K single nucleotide polymorphism (SNP) chip provides an opportunity to increase precision of QTL mapping by increasing genome coverage and map resolution [12]. Using this chicken 60K SNP chip, some QTLs and candidate genes affecting chicken growth have been identified at 172–175 Mb on GGA1 [13] and 71.6–80.2 Mb on GGA4 [14].

The aim of this study was to identify QTLs for the antibody response to NDV in chickens. A genome-wide association study (GWAS) was performed using the chicken 60K SNP chip, based on the data from an F_2_ resource population that was derived from the cross between two broiler lines with differing resistances.

## Results

### Characterization of the antibody response to NDV in the F_2_ population

The antibody response to NDV was positive in all 511 F_2_ birds at 41 days after the second immunization, as assessed by an enzyme linked immunosorbent assay (ELISA) test, and were highly variable. The mean ± standard error of the adjusted antibody responses to NDV was 3.38 ± 0.05, its coefficient of variance was 34.0%, and its distribution fitted a normal distribution (*P*=0.5527, Additional file 1: Figure S1). The heritability of the secondary antibody response to NDV was estimated as 0.24 ± 0.08.

### Genome-wide association analysis

**Figure 1:**
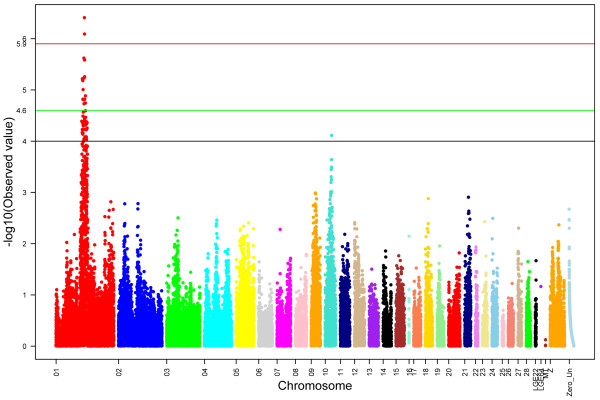
**Manhattan plot of genome**-**wide association analysis for the antibody response to the Newcastle disease virus.** The black line represents a *P* value of 10^-4^, the green line indicates genome-wide significance of suggestive association (*P*<2.51×10^-5^), and the red line indicates genome-wide 5% significance, with a *P*-value threshold of 1.26×10^-6^.

According to the quality control criteria, 505 out of 511 birds in the F_2_ population and 39833 out of 57636 SNPs on the Illumina 60K Chicken SNP Beadchip were eligible for inclusion in the genome-wide association analysis. The informative SNPs in this GWAS were distributed across the chicken genome, at an average interval of 26.4 Kb (Additional file 2: Table S1). The global view of *P* values for all SNPs affecting the antibody response to NDV showed that a region on GGA1 was strongly associated with the chicken antibody response to NDV at 41 days after the second immunization (Figure 1). Two SNP effects reached 5% Bonferroni genome-wide significance (*P*<1.26×10^-6^): *rs15354805* at 100399530 bp of GGA1 and *rs15355555* at 100994585 bp of GGA1. SNPs *rs15354805* and *rs15355555* explained 5% and 4% of the phenotypic variation in antibody response to NDV at 41 days after the second immunization in the F_2_ population, respectively (Table 1). Fourteen SNPs with suggestive association (*P*<2.51×10^-5^) covered a region of approximately 12.2 Mb from 92.2 Mb to 104.4 Mb on GGA1. All the SNPs with *P* values smaller than 1.00×10^-4^ were concentrated in the 89.7–111.0 Mb region of GGA1, except for one SNP on GGA10 (Additional file 3: Table S2). Collectively, all the SNPs with *P*<1.00×10^-4^ explained 8% of the phenotypic variation in the antibody response to NDV, whereas just two SNPs, *rs15354805* on GGA1 and *rs14008095* on GGA10, accounted for 7% of this phenotypic variation.

**Table 1:** **Genome**-wide significant* **SNPs for antibody response to the Newcastle disease virus**

**SNP ID**	**GGA**	**Position(bp)**	**Nearest gene**	**SNP**	**FA**	**FAW**	**FAR**	**FAS**	***P*value**	**R**^**2**^
*rs15354805*	1	100399530	*ROBO2*	C/T	C	0.756	0.750	1.000	3.90×10^-7^	0.05
*rs15355555*	1	100994585	243 Kb upstream of *ROBO2*	A/G	G	0.758	0.750	1.000	8.15×10^-7^	0.04

Note: ‘FA’=favorable allele with higher antibody response to the Newcastle disease virus, ‘FAW’= favorable allele frequency of the F_2_ population, ‘FAR’= favorable allele frequency of the fast-growing Chinese yellow broiler sire line, ‘FAS’= favorable allele frequency of the Huiyang beard chicken. *Significant at the 5% level by the Bonferroni correction.

### Evaluation of the candidate region comprising *rs15354805* and *rs15355555*

**Figure 2:**
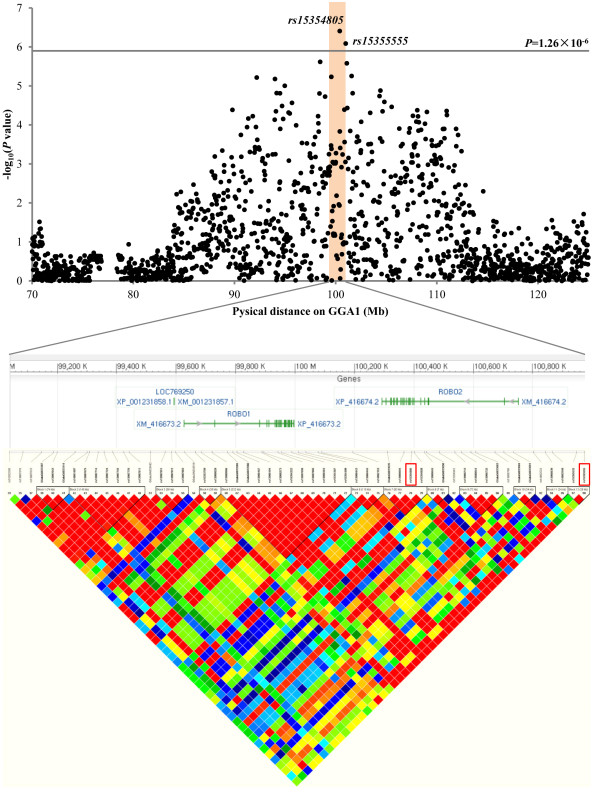
**Genome scan for antibody response to the Newcastle disease virus on chicken (*Gallus gallus*) chromosome 1 with *rs15354805 *and *rs15355555 *SNPs.** The black line indicates genome-wide 5% significance, with a *P*-value threshold of 1.26×10^-6^. Adjusted *P* values on a log_10_ scale for SNPs examined for their association with antibody response to the Newcastle disease virus. Structures, orientations and locations of the *LOC769250*, *ROBO1* and *ROBO2* genes on the NCBI Reference Sequence Build 2.1 are shown, together with pairwise LD estimates and haplotype blocks of the SNPs located within a 2-Mb region with *rs15354805* and *rs15355555* SNPs (red box).

Strong linkage disequilibrium (LD) in GGA1 occurred between SNP markers within a distance of approximately 200 Kb, and the LD declined to the background level (r^2^=0.1) at a distance of about 2 Mb in the F_2_ population (Additional file 4: Figure S2). As shown in Figure 2, there are 12 haplotype blocks in the 99–101 Mb region of GGA1, including *rs15354805* and *rs15355555*. This region contains three protein coding genes: *LOC769250*, *Roundabout*, *axon guidance receptor*, *homolog 1* (*ROBO1*) and *Roundabout*, *axon guidance receptor*, *homolog 2* (*ROBO2*). *Rs15354805* and three additional SNPs (*GGAluGA033635*, *rs13908604* and *rs15354828*) formed an LD block at a distance of 92 Kb in the F_2_ population. All four SNPs are in *ROBO2*, and *rs15354805* is located in intron 7 of chicken *ROBO2*. Although *rs15354805* and *rs15355555* are in an approximately 600 Kb interval, and are not in the same haplotype block, the pairwise LD, as represented by the r-square value between *rs15354805* and *rs15355555*, reached 0.98. SNP *rs15355555* is located about 243 Kb upstream of *ROBO2*. This result suggested that the same underlying genetic factor caused *rs15354805* and *rs15355555* to be significantly associated with the antibody response to NDV.

### Bioinformatic analysis of candidate regions

As shown in Additional file 3: Table S2, there are 194 genes or non-coding RNAs in the candidate regions of 1-Mb windows (SNP position ± 0.5 Mb) surrounding each selected SNP (*P*<1.00×10^-4^). These genes participate in at least 25 KEGG pathways, and comprise at least 108 protein domains in the Interpro database of chickens. Most of the genes in the significantly (*P*<0.1) enriched pathways and protein domains are related to the immune system (Additional file 5: Table S3). Among these, *CD2* and *CD80*, which contain nearly significant (*P*<1.00×10^-4^) SNPs (*rs13904539*, *rs13904683* and *GGaluGA032608*) for the antibody response to NDV, belong to the cell adhesion molecules pathway (gga04514), which involves the major histocompatibility complex (MHC) and T cell receptor genes. LOC418424, junction adhesion molecule 2, CD200, CD80, ROBO1 and ROBO2 on GGA1, and Aggrecan on GGA10 all possess a conserved protein domain called the immunoglobulin-like fold (IPR013783, Additional file 5: Table S3), suggesting that these proteins could be members of the complex gene network that modulates the immune response. The *Cbl proto*-*oncogene*, *E3 ubiquitin protein ligase B*, *interleukin 10 receptor*, *beta*, *interferon gamma receptor 1* and *interferon gamma receptor 2* genes, whose proteins participate in the Jak-STAT signaling pathway (gga04630), are also implicated in the antibody response to NDV.

## Discussion

The host immune response to viruses is a complex process. The antibody response to NDV can be considered to be a quantitative trait under polygenic control, but with some QTLs [10,11]. In the current study, GWAS identified a region located approximately 100 Mb from the proximal end of GGA1, in which *rs15354805* was most significantly (*P*<1.26×10^-6^) associated with, and accounted for 5% of the phenotypic variation of, the antibody response to NDV (Table 1). Moderately high heritability (0.41 ± 0.17) of the chicken antibody response to NDV was observed three decades ago [15]. Heritabilities of the primary antibody response to NDV two weeks post-vaccination were 0.27 ± 0.06 and 0.29 ± 0.05 in two Tanzania chicken ecotypes (*Kuchi* and Tanzania Medium), respectively [16]. Lwelamira also reported recently that the heritability of the chicken primary antibody response to NDV at two weeks post-immunization was 0.22 ± 0.08 in the offspring of *Kuchi* chickens [17], a figure comparable to the 0.24 ± 0.08 estimated here for the secondary antibody response. The QTL linked to *rs15354805* therefore accounted for >20% (0.05/0.24) of the additive genetic variance of this trait. It is reasonable to consider that this QTL would be important for improving the ability of the immune response to NDV in chickens by use of marker-assisted selection. In addition, the effect of combining *rs15354805* on GGA1 and *rs14008095* on GGA10 is almost same as the sum of the effects of all SNPs with *P* values less than 1.00×10^-4^ for the antibody response to NDV (7% vs. 8% of the phenotypic variation) in this study. On that basis, there are probably only two divergent QTLs for this trait on GGA1 and GGA10, while other SNP effects might result from LD.

It should be noted that the region found in the present study to be significantly (*P*<1.26×10^-6^) associated with the antibody response to NDV differs from those found in earlier reports [10,11]. Yonash et al. reported that QTLs for the antibody response to NDV were located on GGA2 and GGA18 [11], whereas Biscarini et al. found 13 QTLs associated with the antibody response to NDV on GGA3, GGA4, GGA5, GGA9, GGA13, GGA16, GGA22 and GGAZ [10]. There are many possible reasons for the differing results among these studies. The population specificity of QTLs for the antibody response to NDV is probably the most important reason [18]. The current study was based on a broiler resource population, while the other two studies were based on nine different laying lines [10], and a chicken resource population built from the intercross of broilers with high and low antibody response to *Escherichia coli* vaccine [11]. In addition, Yonash et al. focused on the primary antibody response to NDV [11], while the current study examined the secondary antibody response to NDV. In the primary antibody response, the predominant class of antibody produced is immunoglobulin M, and in the secondary antibody response it is immunoglobulin Y; therefore, the ranking of birds on antibody response to the primary immunization may differ from that after the secondary boosting resulting in different QTLs being detected. In fact, the QTLs for the antibody response to NDV in this study may reflect the ability of the memory cell pool to respond to NDV. Interestingly, we observed that certain immunity-related genes, such as *CD2*, *CD80* and *CD200*, on the detected QTLs (Additional file 3: Table S2). However, some detected QTLs in the current study were similar to those in two other studies, if the significance threshold set as *P*<0.05 (Figure 1). For example, *rs14520447* (GGA5: 21181095 bp, *P*=0.0031), *rs14520528* (GGA5: 21329838 bp, *P*=0.0194) and *GGAluGA278144* (GGA5: 21355363 bp, *P*=0.0194) were to the strongest of the QTLs detected by Biscarini et al. [10], and *rs10727400* (GGA18: 4572183 bp, *P*=0.0336), *rs15821136* (GGA18: 4587942 bp, *P*=0.0171) and *GGAluGA120237* (GGA18: 4594180 bp, *P*=0.0200) were similar to the QTLs detected on GGA18 in the report of Yonash et al. [11].

The MHC plays an important role in animal immunity [19-22]. However, the present study found that the most reliable QTL linked to antibody response to NDV could be in the region that included *rs15354805* and *rs15355555* on GGA1, whereas it did not detect any significant SNPs on GGA16, which harbors the chicken MHC. This may be because very few SNP markers on GGA16 could be used for QTL mapping in the current study (Additional file 2: Table S1). Further studies are necessary in order to detect QTLs for the antibody response to NDV on GGA16.

The 2 Mb region of 99–101 Mb region containing three coding genes on GGA1 had the most significant effects for the secondary antibody response to NDV (Figure 2). Within this region, *LOC769250* is a predicted gene with no clear function, and *ROBO1* and *ROBO2* belong to the *ROBO* family, which are members of the immunoglobulin superfamily [23]. The encoded products of both genes are integral membrane protein receptors for the SLIT-family, and have similar functions in axon guidance and migration of neuronal precursor cells [24-27]. Many molecules, such as Semaphorin 7A, Neurokinin A and Plexin-B2, participate in nervous system and immune system processes [28-32]. Perhaps ROBO1 and ROBO2 also affect the immune system. In fact, SLIT/ROBO signaling can influence the expression of Rac1 and CDC42 [33]. Rac1 plays a crucial role in T cell development [34] while CDC42 is essential for memory T cell growth and differentiation [35,36]. Therefore, SLIT/ROBO signaling could contribute to adaptive immunity through Rac1 or/and CDC42. Consistently, the focus of QTL mapping in this study was the difference of secondary antibody response to NDV that would be mainly related to activation, growth and differentiation of the memory T cells after the second immunization. In addition, SLIT/ROBO signaling plays important roles in leukocyte chemotaxis [37-39]. Therefore, *ROBO1* and *ROBO2* could well be candidate genes for modulating the antibody response to NDV. However, determining the functional role of these QTLs in the antibody response to NDV needs requires further studies. Studies of chicken network pathways involving SLIT/ROBO signaling need to be replicated to validate the present findings.

In addition to *ROBO1* and *ROBO2*, some of the other genes that are close to the QTL region (*P*<1.00×10^-4^, Additional file 3: Table S2) in the current study might modulate antibody responses to NDV, based on their biological function and networks. These genes could comprise a complex network (Additional file 6: Figure S3). On GGA10, *interleukin 16*, whose encoded protein is linked to ROBO2 by three pathways (Additional file 6: Figure S3), participates in leukocyte chemotaxis and positive regulation of responses to external stimuli [40], while B-cell CLL/lymphoma 2 - related protein A1 is essential for lymphocyte activation and cell survival [41]. In fact, seven genes in the QTL region on GGA1 were enriched in the cell adhesion molecules pathway, which affects the immune system (Additional file 5: Table S3). Among these genes, *CD2* and *CD80* were initially considered to be candidate genes. CD2 not only plays an important role in the generation of nanotubes between natural killer cells and their targets [42], but also provides signals to control the development and differentiation of T cells [43-45]. CD80 is expressed on B cells, but also induces T cell proliferation and cytokine production [46-48]. In addition, CD200, which is about 180 Kb from *rs14856616* (*P*=6.08×10^-6^), is expressed on the surface of immune cells and affects the adaptive immune system by stimulating proliferation of T cells [49]. Interestingly, evidence shows that dendritic cells and T cells show a stronger response in the absence of CD200 inhibitory signaling [50]. In addition, there is a relationship between CD200 and influenza virus infection [51]. SAM domain, SH3 domain and nuclear localization signals 1 is another immune-related protein that participates in B cell activation and differentiation [52]. CD247, IFNGR2 and IFNAR1 belong to the natural killer cell mediated cytotoxicity pathway (Additional file 5: Table S3). Obviously, a large number of genes related to innate and adaptive immunity are in the 89.7-111.0 Mb region of GGA1. This QTL region also overlaps the QTL for the antibody response to sheep red blood cells [53]. Thus this genomic region is of great importance for the chicken immune response and, possibly, disease resistance, and has been impacted by intense artificial selection (e.g., ZH_p_=−6.2 in *ROBO2*) during chicken domestication [54].

## Conclusions

This study has exposed a novel QTL region associated with the antibody response to NDV. The QTL region was located approximately 100 Mb from the proximal end of GGA1. This region may play an important role in the chicken immune response. *ROBO1* and *ROBO2* were found to be important prospective candidate genes for modulating the antibody response to NDV in chickens; however, the roles of *ROBO1* and *ROBO2* require further study, such as exploring the effects of *ROBO1* and/or *ROBO2* silencing and overexpression in vitro and in vivo on immune gene networks.

## Methods

### Ethics statement

The Animal Care Committee of the Institute of Animal Science, Guangdong Academy of Agricultural Sciences (Guangzhou, P. R. China) approved this study (approval number GAAS-IAS-2009-73). Animals involved in the current study were humanely euthanized, as necessary, to reduce any suffering.

### Experimental population

The F_2_ resource population was built from the full-sib intercross of two divergent lines (23 P and 51 F_1_). The first line was a fast-growing Chinese yellow broiler raised at Guangdong Wiz Agricultural Science & Technology Co. Ltd., which had undergone more than ten generations of selection for high growth rate and meat quality tailored to Chinese tastes. The second line was the Huiyang beard chicken, a Chinese local breed with a low growth rate and high meat quality tailored to Chinese tastes. The resistance of the second line is better than that of the first line (Data not shown). The F_2_ population comprised 511 individuals from six hatches. Before 40 days of age, each hatch was maintained in a group cage. From 40 days to 91 days, each chicken was reared in an individual cage with an individual trough. The house was equipped with water curtain systems and supplied with 24-hour light per day. A starter diet (200 g total protein and 2,900 kcal ME/kg) from hatch to 35 days of age and a grower diet (180 g total protein and 2,950 kcal ME/kg) from 43 days to 91 days of age were provided ad libitum.

### Phenotypic measurements

The chickens were immunized with a commercial NDV live vaccine of the LaSota strain (Intervet International B.V., Boxmeer, Netherlands), using the standard dose given in the instructions of the vaccine, by eye drop at 25 days of age and at 50 days of age, respectively. At 41 days after the second immunization (91 days of age), serum samples were collected from the chickens. The antibody response to NDV was determined using an indirect ELISA and expressed as the S/P value of corresponding dilutions, according to the instruction of the commercial ELISA kit for testing the antibody response to NDV (BioCheck, Inc., Foster City, CA, USA). The ELISA kit measures NDV-specific immunoglobulin, Y but not immunoglobulin A nor immunoglobulin M.

### Genotyping procedures

Genomic DNA extraction from venous blood was performed using a phenol/chloroform method. For all 511 F_2_ individuals, the quality and concentration of genomic DNA fulfilled the requirements of the Illumina Infinium SNP genotyping platform. Genotyping using the Illumina 60K Chicken SNP Beadchip [12] was carried out at the Illumina-certified service provider, DNA LandMarks Inc., Saint-Jean-sur-Richelieu, Quebec, Canada. Quality control was assessed in GenomeStudio v2008.1 [55]. Six samples were excluded because they had more than 5% missing SNP genotypes. The final SNP set used in this study consisted of 39,833 SNP markers, meeting the following selection criteria: call frequency >95%, heterozygosity cluster intensity and separation value >0.4, and minor allele frequency >0.1. Information about the SNPs on each chromosome is summarized in Additional file 2: Table S1. Gene locations were based on the Ensembl Genome Browser [56] and the National Center for Biotechnology Information (NCBI) [23].

### Data analysis

Variance components were estimated using the average information restricted maximum likelihood algorithm [57] implemented by the DMU package [58]. The model was as follows:

y=Xb+Za+e

where y is the vector of observations of antibody response to NDV; b is the vector of fixed effects including sex (two levels) and hatch (six levels); a is the vector of animal additive genetic effects; e is the vector of random residuals; X and Z are corresponding incidence matrices. The variance matrices of random effects were $\mathrm{var}\left(a\right)=G=A\sigma_{a}^{2}$ and $\mathrm{var}\left(e\right)=R=I\sigma_{e}^{2}$, and the distributions for the random effects were assumed to be:

$$a\sim N\left(O,G\right)\text{and}\;e\sim N\left(O,G\right),$$

where A is the additive relationship matrix between individuals in the pedigree of all birds; $\sigma_{a}^{2}$ is animal additive genetic variance; I is an identity matrix; and $\sigma_{e}^{2}$ is the residual variance. Heritability of antibody response to NDV was estimated as $h^{2}=\frac{\sigma_{\alpha }^{2}}{\sigma_{p}^{2}}=\frac{\sigma_{\alpha }^{2}}{\sigma_{\alpha }^{2}+\sigma_{e}^{2}}$, and $R^{2}=\frac{\sigma_{\mathrm{QTL}}^{2}}{\sigma_{p}^{2}}$, thus, the ratio of the QTL effect compared with the additive genetic variance was calculated as $Q^{2}=\frac{\sigma_{\mathrm{QTL}}^{2}}{\sigma_{a}^{2}}=\frac{R^{2}}{h^{2}}$, where $\sigma_{p}^{2}$, $\sigma_{\mathrm{QTL}}^{2}$ and R^2^ are the phenotypic variance, the QTL effect variance, and coefficient of correction for the regression equation with one or more QTL as independent variable and the antibody response to NDV as a dependent variable, which indicated the ratio of the QTL effect compared with the phenotypic variance of antibody response to NDV, respectively.

A two-step GWAS procedure was performed in this study. First, the phenotypic data in the F_2_ population were adjusted for sex, hatch, sire and dam effects by the following model:

$$Y_{\mathrm{ijkl}}=\mu +{\text{SEX}}_{i}+H_{j}+S_{k}+D_{\mathrm{kl}}+e_{\mathrm{ijkl}}$$

where Y_ijkl_ is the phenotypic value for the antibody response to NDV, *μ* is the overall mean, SEX_i_ is the effect of the i^th^ sex, H_j_ is the effect of the j^th^ hatch, S_k_ is the effect of the k^th^ sire, D_kl_ is the effect of the l^th^ dam within k^th^ sire, and e_ijkl_ is the residual effect. The adjusted phenotypic value was calculated as *μ* + e_ijkl_.

Second, the adjusted antibody response to NDV, which followed a normal distribution (Additional file 1: Figure S1), was used to carry out genome-wide association analysis in PLINK using linear regression analyses without any covariates other than SNPs [59]. The threshold *P* value of the 5% Bonferroni genome-wide significance was 1.26×10^-6^ (0.05/39833), and the threshold *P* value for the “suggestive linkage” significance, which allowed one false positive effect in a genome-wide test, was 2.51×10^-6^ (1/39833). Using the “gap” package in R v2.12.0 (http://www.r-project.org), a Manhattan plot was drawn to describe the *P* values of all SNPs. This study calculated the fraction of the phenotypic variance explained by the significantly associated SNPs according to a previously described method [60]. Haploview software [61] was used to calculate LD as an r-square value and build haplotype blocks under the four gamete rule (the 4^th^ gamete must be observed at frequency >0.01) in the candidate region associated with antibody response to NDV in the F2 population.

### Bioinformatics analysis

Names of genes in the candidate regions with 1-Mb windows (SNP position ± 0.5 Mb) surrounding each significant SNP (*P*<1.00×10^-4^) were obtained from the Ensembl Genome Browser [56] and the NCBI [23]. A pathway and protein domain analysis was performed using the Database for Annotation, Visualization and Integrated Discovery (DAVID) [62,63]. A network of gene correlation was constructed by integrating the pathways from the human database of the Kyoto Encyclopedia of Genes and Genomes (KEGG) [64] (http://www.genome.jp/kegg/), the Gene Map Annotator and Pathway Profiler (GenMAPP) [65] (genmapp.org/) and the BioCarta (http://www.biocarta.com/genes/index.asp).

## Competing interests

The authors have no competing interests to declare.

## Authors’ contributions

CL, HQ, XH, NL and DS conceived and designed the experiments; CL, HQ, JM, JW, CYL, CY and DS performed the experiments; CL analyzed the data; JM, CY, NL and DS contributed the materials; CL and HQ wrote the paper; and CL, HQ and DS obtained the funding. All authors read and approved the final manuscript.

## Supplementary Material

Additional file 1: Figure S1 Distribution of the adjusted antibody response to the Newcastle disease virus in the F2 population. The data fitted the normal distribution (Shapiro-Wilk W test *P*=0.5527). Click here for file

Additional file 2: Table S1 SNP markers used in the genome-wide association study. Click here for file

Additional file 3: Table S2 SNPs associated with antibody response to Newcastle disease virus (*P*<1.00×10^-4^). Click here for file

Additional file 4: Figure S2 Pattern of linkage disequilibrium on chicken (*Gallus gallus*) chromosome 1. Click here for file

Additional file 5: Table S3 Functional annotation of candidate genes by KEGG and Interpro analysis in chickens (*P*<0.1). Click here for file

Additional file 6: Figure S3 Network of gene correlation for genes in candidate regions related to antibody response to Newcastle disease virus (*P*<1.00×10^-4^). Yellow ellipses indicate genes. Green lines, red lines and blue lines indicate pathways based on GenMAPP, KEGG and BioCarta, respectively. The numbers indicate the number of pathways related to the two genes. Click here for file
