# Supplementary figures and images for: Genome-wide association study of antibody response to Newcastle disease virus in chicken
Source: BMC Genet. 2013 May 10;14:42. doi: 10.1186/1471-2156-14-42 (PMC3654938; doi:10.1186/1471-2156-14-42)

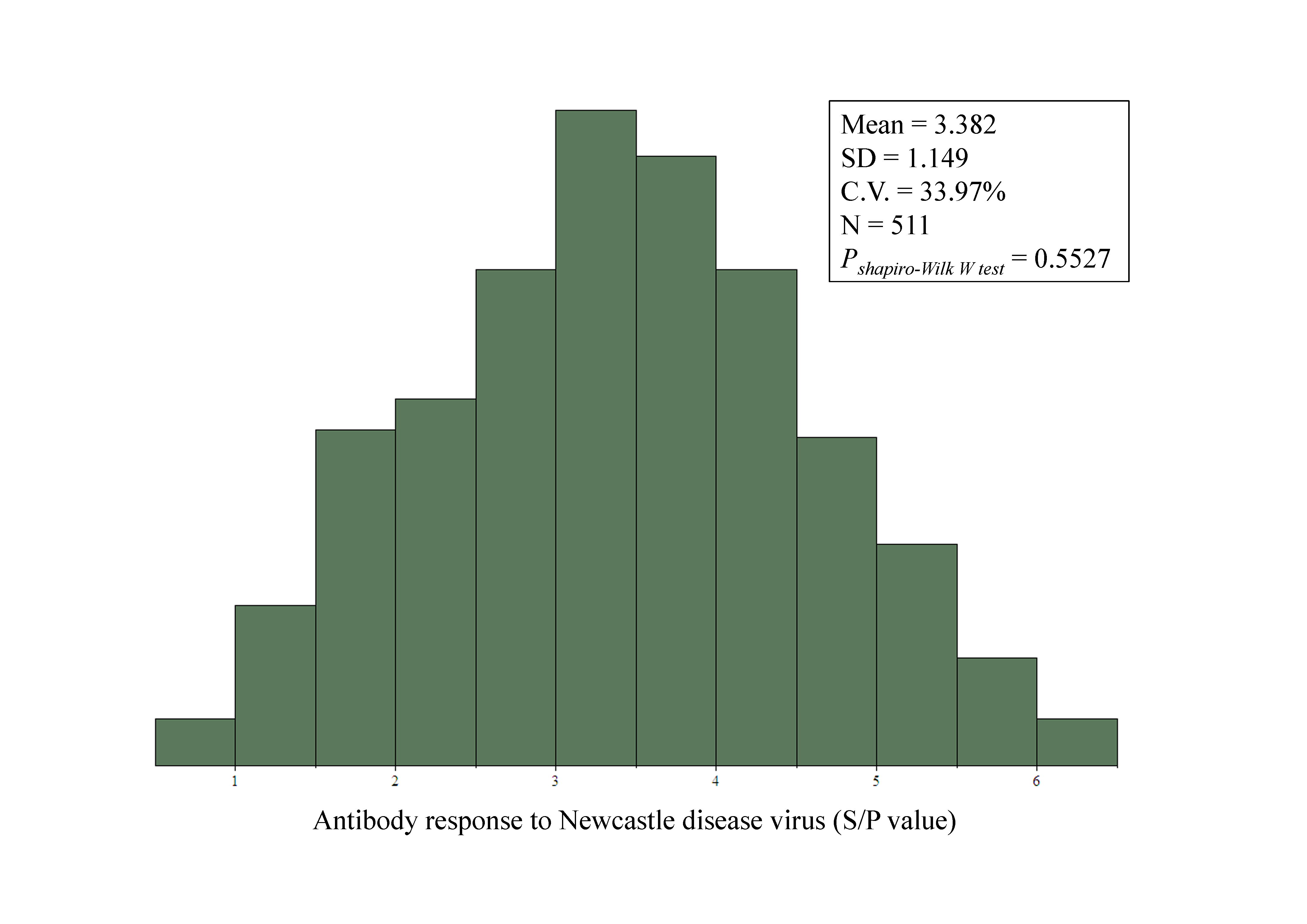

Supplement: Additional file 1: Figure S1 — Distribution of the adjusted antibody response to the Newcastle disease virus in the F2 population. The data fitted the normal distribution (Shapiro-Wilk W test P=0.5527). [file 1471-2156-14-42-S1.tiff]

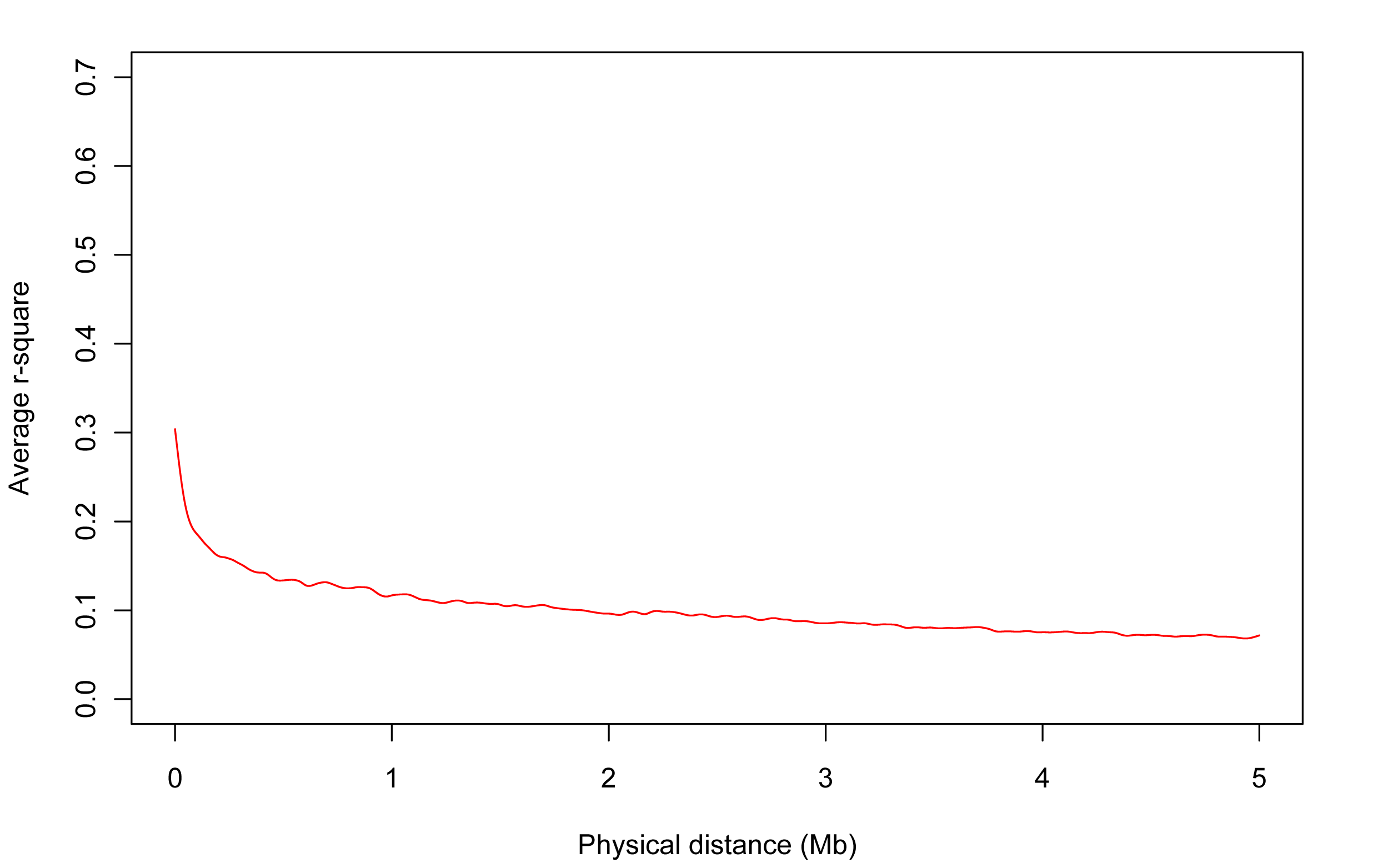

Supplement: Additional file 4: Figure S2 — Pattern of linkage disequilibrium on chicken (Gallus gallus) chromosome 1. [file 1471-2156-14-42-S4.tiff]

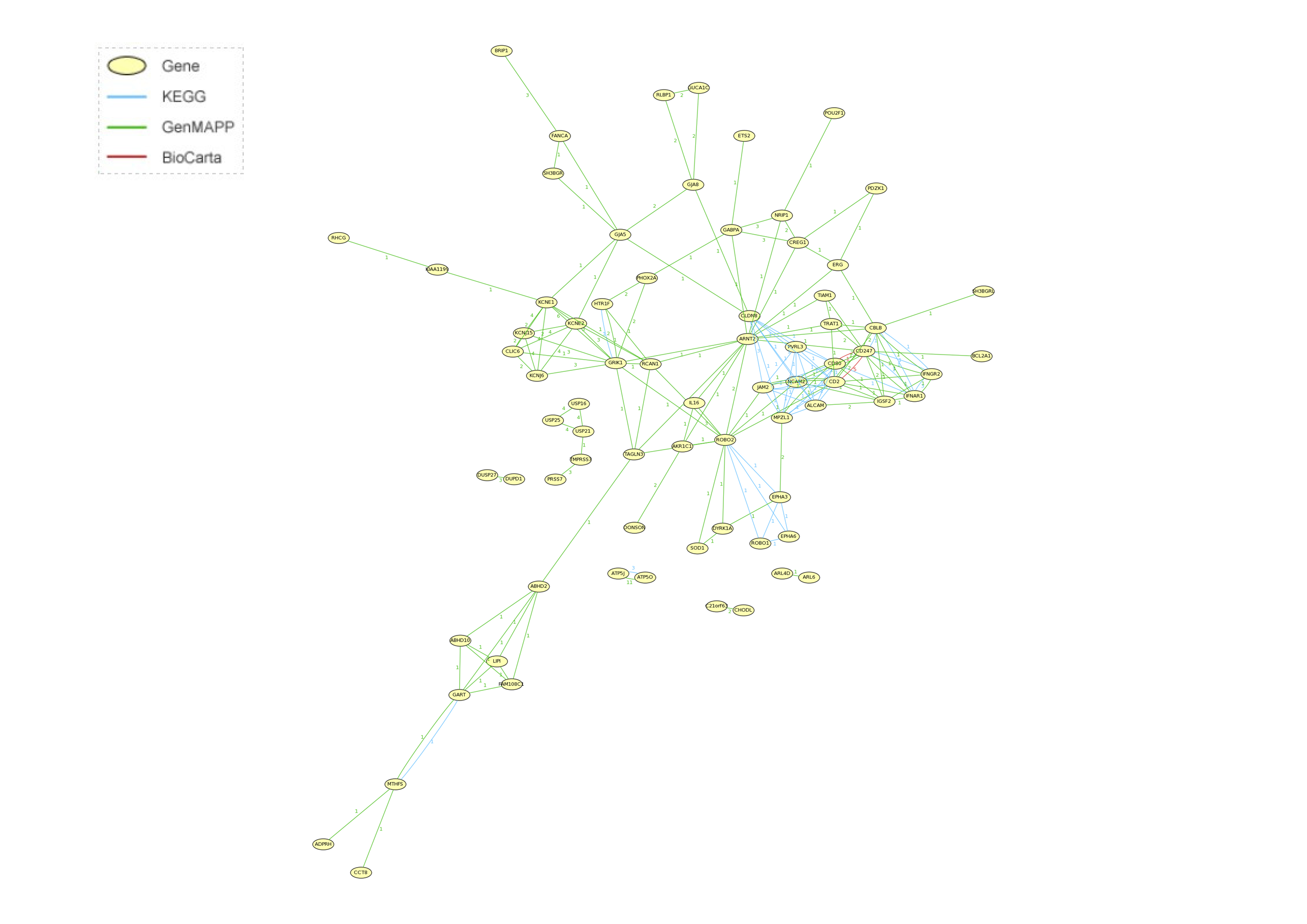

Supplement: Additional file 6: Figure S3 — Network of gene correlation for genes in candidate regions related to antibody response to Newcastle disease virus (P<1.00×10^-4^). Yellow ellipses indicate genes. Green lines, red lines and blue lines indicate pathways based on GenMAPP, KEGG and BioCarta, respectively. The numbers indicate the number of pathways related to the two genes. [file 1471-2156-14-42-S6.tiff]
